# Genetic Defects in *DNAH2* Underlie Male Infertility With Multiple Morphological Abnormalities of the Sperm Flagella in Humans and Mice

**DOI:** 10.3389/fcell.2021.662903

**Published:** 2021-04-23

**Authors:** Jae Yeon Hwang, Shoaib Nawaz, Jungmin Choi, Huafeng Wang, Shabir Hussain, Mehboob Nawaz, Francesc Lopez-Giraldez, Kyungjo Jeong, Weilai Dong, Jong-Nam Oh, Kaya Bilguvar, Shrikant Mane, Chang-Kyu Lee, Christopher Bystroff, Richard P. Lifton, Wasim Ahmad, Jean-Ju Chung

**Affiliations:** ^1^Department of Cellular and Molecular Physiology, Yale School of Medicine, Yale University, New Haven, CT, United States; ^2^Department of Biotechnology, Faculty of Biological Sciences, Quaid-i-Azam University, Islamabad, Pakistan; ^3^Department of Genetics, Yale School of Medicine, Yale University, New Haven, CT, United States; ^4^Department of Biomedical Sciences, Korea University College of Medicine, Seoul, South Korea; ^5^Department of Biochemistry, Faculty of Biological Sciences, Quaid-i-Azam University, Islamabad, Pakistan; ^6^Yale Center for Genome Analysis, Yale University, New Haven, CT, United States; ^7^Department of Agricultural Biotechnology, College of Agriculture and Life Sciences, Seoul National University, Seoul, South Korea; ^8^Designed Animal and Transplantation Research Institute, Institutes of Green Bio Science and Technology, Seoul National University, Pyeongchang-gun, South Korea; ^9^Department of Biological Sciences, Rensselaer Polytechnic Institute, Troy, NY, United States; ^10^Laboratory of Human Genetics and Genomics, The Rockefeller University, New York, NY, United States; ^11^Department of Obstetrics, Gynecology and Reproductive Sciences, Yale School of Medicine, Yale University, New Haven, CT, United States

**Keywords:** male infertility, asthenozoospermia, WES, *DNAH2*, sperm flagellum, MMAF

## Abstract

Asthenozoospermia accounts for over 80% of primary male infertility cases. Reduced sperm motility in asthenozoospermic patients are often accompanied by teratozoospermia, or defective sperm morphology, with varying severity. Multiple morphological abnormalities of the flagella (MMAF) is one of the most severe forms of asthenoteratozoospermia, characterized by heterogeneous flagellar abnormalities. Among various genetic factors known to cause MMAF, multiple variants in the *DNAH2* gene are reported to underlie MMAF in humans. However, the pathogenicity by DNAH2 mutations remains largely unknown. In this study, we identified a novel recessive variant (NM_020877:c.12720G > T;p.W4240C) in *DNAH2* by whole-exome sequencing, which fully co-segregated with the infertile male members in a consanguineous Pakistani family diagnosed with asthenozoospermia. 80–90% of the sperm from the patients are morphologically abnormal, and *in silico* analysis models reveal that the non-synonymous variant substitutes a residue in dynein heavy chain domain and destabilizes DNAH2. To better understand the pathogenicity of various *DNAH2* variants underlying MMAF in general, we functionally characterized *Dnah2*-mutant mice generated by CRISPR/Cas9 genome editing. *Dnah2*-null males, but not females, are infertile. *Dnah2*-null sperm cells display absent, short, bent, coiled, and/or irregular flagella consistent with the MMAF phenotype. We found misexpression of centriolar proteins and delocalization of annulus proteins in *Dnah2*-null spermatids and sperm, suggesting dysregulated flagella development in spermiogenesis. Scanning and transmission electron microscopy analyses revealed that flagella ultrastructure is severely disorganized in *Dnah2*-null sperm. Absence of DNAH2 compromises the expression of other axonemal components such as DNAH1 and RSPH3. Our results demonstrate that DNAH2 is essential for multiple steps in sperm flagella formation and provide insights into molecular and cellular mechanisms of MMAF pathogenesis.

## Introduction

Infertility is an important public health concern, affecting approximately 15% of all couples worldwide (Esteves et al., 2011; Mascarenhas et al., 2012). Male factor infertility accounts for approximately half of the infertility cases and results from extremely heterogeneous pathogenesis. Asthenozoospermia (ASZ), which is defined by reduced sperm motility (WHO, 2010), is one of the most prevalent cases of male infertility (Curi et al., 2003). Together with the motility issues, ASZ patients often show sperm morphological defects, including morphological abnormalities of the sperm flagella (MMAF) (Ben Khelifa et al., 2014). MMAF is characterized by sperm with abnormal flagellar morphologies, such as absent, short, bent, coiled, and/or irregular caliber flagella. MMAF is mainly caused by genetic mutations; around 20 MMAF-associated genes have been reported (Toure et al., 2020). The pathogenic mechanisms of MMAF, however, vary depending on genetic mutations. The currently reported MMAF-associated genes explain only 30–60% of the MMAF cases in different cohorts (Toure et al., 2020), indicating heterogeneous genetic etiologies of the disorder. A full picture of the genetic basis of MMAF awaits further genetic exploration.

Sperm flagella and cilia share a highly organized and evolutionarily conserved microtubule-based structure called axoneme (Inaba, 2011; Viswanadha et al., 2017). The axoneme is composed of nine pairs of peripheral microtubule doublets, and a central pair of microtubules (“9+2” arrangement). The peripheral microtubule doublets are connected through the nexin-dynein regulatory complex, and A tubules in each microtubule doublet harbor multiprotein-complexed axonemal components, including radial spokes (RS), and inner and outer dynein arms (IDA and ODA, respectively). IDA and ODA are motor protein complexes that regulate flagella and cilia beating by ATP hydrolysis (Viswanadha et al., 2017). Previous studies have suggested that primary ciliary dyskinesia (PCD, MIM: 244400) by mutations in IDA or ODA components may cause male infertility based on the structural similarity of sperm flagella to motile cilia (Sironen et al., 2020). However, variants in MMAF-causing genes encoding IDA or ODA proteins have been identified in infertile males without PCD symptoms. These MMAF-specific mutations suggest that molecular compositions and the workings of IDA and ODA in sperm flagella, which are yet to be well-characterized, might be distinct to those in motile cilia.

Variants encoding ODA proteins [DNAH8 (MIM: 603337; Liu et al., 2020; Yang et al., 2020) and DNAH17 (MIM: 610063; Whitfield et al., 2019; Sha et al., 2020; Zhang B. et al., 2020; Zhang et al., 2021)] and IDA proteins [DNAH1 (MIM: 603332; Ben Khelifa et al., 2014; Amiri-Yekta et al., 2016; Sha Y. et al., 2017; Wang et al., 2017) and DNAH2 (MIM: 603333; Li et al., 2019)] are reported to cause MMAF in human. *DNAH2* encodes a heavy chain of IDA. Among over 30 single nucleotide variants (SNVs) in *DNAH2* annotated in ClinVar, five variants (four heterozygous and one homozygous) are reported to be pathogenic (Li et al., 2019) but lack functional studies in animal models.

Here, we report a new bi-allelic non-synonymous mutation in *DNAH2* identified from a Pakistani consanguineous family with infertile males diagnosed to oligoasthenozoospermia. Using a mouse model, we found DNAH2 deficiency causes male infertility with MMAF phenotypes and aberrant protein expression of various structural components in sperm flagella. Our findings demonstrate that DNAH2 is essential for sperm flagellar development and structural stabilization, providing an insight into the pathogenicity and variable phenotypic severity of DNAH2-associated MMAF.

## Materials and Methods

### Subjects and Family

Present study was approved by the Institutional Review Board of Quaid-i-Azam University, Islamabad, Pakistan (IRB00003532, IRB protocol# QAU-171) and Yale Center for Mendelian Genomics. A survey was conducted in selected regions of Pakistan to document cases of infertility. The family to participate this study were informed about the nature of work and the possible outcomes of the study. Written informed consent was provided by the family.

### Sample Collection and Clinical Investigation

Semen samples were collected from affected individuals after 2–5 days of abstinence from sexual intercourse according to World Health Organization guidelines (WHO, 2010). The samples were analyzed for semen volume, pH, color, and viscosity. Semen samples were subjected to liquefaction at 37°C for 30–60 min. Sperm concentration, motility and morphology were analyzed. For detailed sperm morphology analyses, semen samples were subjected to PAP staining according to protocols approved by WHO (2010). Venous blood from the affected and control members were collected and stored at 4°C. Hormone assay was carried out using automated immunoassay analyzer AIA-360 (Tosoh Bioscience, Inc.). Karyotyping was performed by establishing and harvesting phytohemagglutinin (PHA) after 72 h. Twenty metaphases were then analyzed following Giemsa-Trypsin banding (Howe et al., 2014). Genomic DNA was extracted using QIAamp DNA Mini Kit (Qiagen). To search for the Y-chromosome microdeletions spanning four regions (AZFa, AZFb, AZFc, and AZFd), PCR was performed using different sets of primer pairs.

### Whole-Exome Sequencing and Data Analysis

1.0 μg of blood genomic DNA is sheared to a mean fragment length of about 140 bp using focused acoustic energy (Covaris E210). Exome sequencing was performed by exome capture using the IDT xGen capture probe panel with an additional “spike-in” of ∼2,500 regions, totaling ∼620 kb, of RefGene coding regions that were not included or were poorly covered by the IDT panel. Captured fragments were sequenced using 101 bp paired-end sequencing reads in an Illumina NovaSeq 6000 with a S4 flow cell according to Illumina protocols. Sequencing reads were aligned to human genome build 37 (GRCh37/hg19) using the BWA-MEM (Li, 2013), aggregated into a BAM file, and further processed to produce variants with GATK v3.4 (McKenna et al., 2010) following the GATK Best Practices workflow (van der Auwera et al., 2013). Variants were annotated with ANNOVAR (Wang et al., 2010) and MetaSVM (Dong et al., 2015) was used to predict the deleteriousness of non-synonymous variants. For rare transmitted dominant variants, only loss of function mutations (stop-gains, stop-losses, canonical splice-sites, and frameshift indels) and D-mis mutations (non-synonymous mutations predicted deleterious by MetaSVM) were considered potentially damaging and filtered using the following criteria to reduce false positives: (1) GATK variant quality score recalibration (VQSR) of PASS, (2) MAF ≤ 2 *×* 10^–5^ in the Genome Aggregation Database (gnomAD) v2.1 (Lek et al., 2016)^1^ (calculated based on combined dataset of WES and WGS data from gnomAD database), (3) DP ≥ 8 independent reads, (4) GQ score ≥20, (5) MQ score ≥40, (6) PLdiff/DP ≥8, and (7) indels in Low Complexity Regions (LCRs) were also excluded. Transmitted recessive variants were filtered for rare (MAF ≤ 10^–3^ in gnomAD) homozygous and compound heterozygous variants using the same criteria described above. Candidate variants were confirmed by PCR amplification followed by Sanger sequencing.

### Kinship Analysis

Pairwise proband relatedness and pedigree information of extended family were confirmed using KING v2.2.4 (Manichaikul et al., 2010) by estimating kinship coefficient. Inbreeding coefficient was calculated by homozygosity-by-descent (HBD). The HBD segments in the patients were detected using Beagle v3.3.2 (Browning and Browning, 2011). The criteria of consanguinity are defined as runs of homozygosity in segments of 2 cM or greater length that collectively comprise at least 0.35% of the genome.

### Copy Number Variation Analysis

eXome-Hidden Markov Model (XHMM) (Fromer et al., 2012) was run to call CNVs from WES as previously described. GATK DepthOfCoverage was used to calculate mean read depth per targets from the alignment files. The data were normalized by removing the highest variance principal components (variance >70%) and z scores were calculated from the mean read depths. CNVs were called using the Viterbi Hidden Markov model (HMM) and the quality scores were calculated using the forward-backward HMM. After filtering out common CNVs present at allele frequencies greater than 0.1% in 1000 Genomes and 10% in the cohort, high quality CNVs (SQ > 60 where SQ indicates the phred-scaled quality score for the presence of a CNV event within the interval) were subjected to visual inspection.

### Genomic DNA PCR

Genomic DNA PCR was performed to confirm the co-segregating variants screened from WES analyses in the family. Genomic DNA extracted from human blood samples were applied for PCR using OneTaq^®^ 2X Master Mix (NEB) with the primer pairs listed in Supplementary Table 1. The primer pairs were chosen to amplify genomic region containing screened variants. PCR products were gel-purified and Sanger sequenced using the primers for the targeted PCR amplifications.

### Protein Sequence Conservation Analysis

In order to analyze conservation of the mutated residue in each gene, protein sequences of the orthologs were aligned using Clustal Omega (Madeira et al., 2019).

### Sequence-Structure Analysis

Sequence analysis was carried out using web-based tools from the National Center for Biological Information (NCBI), including Genome Database Viewer. Phylogenetic analysis and multiple sequence alignments were carried out in the UGENE (Bykova et al., 2016) software package, using PHYLIP (Retief, 2000) and MUSCLE (Edgar, 2004), respectively. Molecular modeling, including homology-based modeling of mutant sequences, was carried out within the software package MOE (Qiao and Guo, 2005) using the AMBER10:EHT force field (Wang et al., 2004), Images were made using Molecular Operating Environment (MOE, Chemical Computing Group Inc.).

### Single-Cell RNA-seq Analysis

The raw count matrices for human (GSE109037) and mouse (GSE109033) testis single cell RNA (scRNA)-seq datasets (Hermann et al., 2018) were downloaded from Gene Expression Omnibus (GEO) database^2^. The downloaded raw count matrices were processed for quality control using the Seurat package (ver.3.2.3) (Stuart et al., 2019). Briefly, cells with less than 200 expressed features, higher than 9,000 (GSE109033) or 10,000 (GSE109037) expressed features and higher than 20% (GSE109037) or 25% (GSE109033) mitochondrial transcript fraction were excluded to select single-cells with high quality mRNA profiles. The data was normalized by the total expression, scaled and log transformed. Identification of 2,000 highly variable features was followed by PCA to reduce the number of dimensions representing each cell. Statistically significant 15 PCs were selected based on the JackStraw and Elbow plots and provided as input for constructing a K-nearest-neighbors (KNN) graph based on the Euclidean distance in PCA space. Cells were clustered by the Louvain algorithm with a resolution parameter 0.1. Uniform Manifold Approximation and Projection (UMAP) was used to visualize and explore cluster data. Marker genes that define each cluster were identified by comparing each cluster to all other clusters using the MAST (Finak et al., 2015) provided in Seurat package. In order to correct batch effects among samples and experiments, we applied the Harmony package (ver.1.0) (Korsunsky et al., 2019) to the datasets. The Markov Affinity-based Graph Imputation of Cells (MAGIC) algorithm (ver.2.0.3) (van Dijk et al., 2018) was used to denoise and the count matrix and impute the missing data. In these testis datasets from adult human, we identified 23,896 high quality single cells, that were clustered into seven major cell types, including spermatogonia, spermatocytes, early spermatids, late spermatids, peritubular myoid cell, endothelial cell, and macrophage. Similarly, we exploited 30,268 high quality single cells from eight adult and three 6-day postpartum mouse testis tissue samples and subsequently defined 11 major cell populations.

### Animals

Mice were treated in accordance with guidelines approved by the Yale Animal Care and Use Committees that reviewed the animal study protocol (20079). Wild-type (WT, C57BL/6) mice were purchased from Charles river laboratories and *Dnah2*-mutant mice (C57BL/6N-Dnah2^em1(IMPC)Tcp^) were purchased from The Center for Phenogenomics. Founders were generated by using CRISPR/Cas9 technique with two guide RNAs (guide RNA1: 5′- GAGTCACACTAACCACCCCA-3′, guide RNA2: 5′- ATCACTACTCACGGTCACAA-3′) and maintained on a C57/B6 background. Genomic DNA from hetero- and homozygous *Dnah2* mutant mice were used for genotyping with PCR (WT allele, forward: 5′-CTTGTATGCACACCTGCCTTA-3′, reverse: 5′-CATTTTCAATGTTTCAGCCTCACT-3′; mutant allele, forward: 5′-ATGCACACCTGCCTTAACTC-3′, reverse: 5′-ACGACACGCTTCTTCTTTGT-3′). Mice were treated with guidelines approved by the Yale Animal Care and Use Committees.

### Histological Analyses

Testis, epididymis, trachea, and oviduct were collected from *Dnah2* mutant mice and rinsed with PBS. Rinsed tissues were fixed with 4% PFA in PBS for overnight at 4°C. Fixed tissues were washed in PBS and dehydrated by serial incubations in ethanol to 100%. Dehydrated tissues were embedded into paraffin and sectioned. The sections were deparaffinized and stained with hematoxylin and eosin (H/E). Stained sections were imaged with ac1300–200 μm CMOS camera (Basler AG) equipped in Nikon E200 microscope under 10x phase contrast objective (CFI Plan Achro 10X/0.25 pH1 WF, Nikon).

### Sperm Preparation

Epididymal mouse spermatozoa were prepared as described in our previous study (Hwang et al., 2019). Briefly, epididymal sperm were collected in M2 medium (EMD Millipore) or HS medium (135 mM NaCl, 5 mM KCl, 1 mM MgSO_4_, 2 mM CaCl_2_, 20 mM HEPES, 5 mM Glucose, 10 mM Lactic acid, and 1 mM sodium pyruvate, pH 7.4) by swim-out method at 37°C for 10 min. Collected sperm were washed and used for the required experiment.

### Mouse Sperm Motility Analysis

Non-capacitated epididymal sperm cells were transferred to 37°C chamber (Delta T culture dish controller; Bioptechs) filled with HS medium. Sperm motility were recorded for 4 s with 100 fps using Axio observer Z1 microscope (Carl Zeiss) equipped with a high-speed pco.dege sCMOS camera.

### Antibodies and Reagents

Rabbit polyclonal DNAH1 (PA5-57826), DNAH2 (PA5-64309), and DNAH9 (PA5-45744) antibodies were purchased from Thermo Fisher Scientific. Rabbit polyclonal SEPTIN4 antibody (NBP1-90093) was purchased from Novus Biologicals. Monoclonal acetylated tubulin (clone 6-11B-1, T7451), CENTRIN1 (clone 20H5, 04-1624), and γ-tubulin (clone GTU-88, T6557), and rabbit polyclonal SEPTIN12 (HPA041128) antibodies were purchased from MilliporeSigma. In-house rabbit polyclonal RSPH3 antibody was generated in this study by immunizing rabbit with synthesized RSPH3 peptide (TAEASGLYTYSSRPR, Open Biosystems). Antisera from the immunized rabbits were affinity-purified using the peptide immobilized Amino Link Plus resin (Pierce). Goat anti-mouse or rabbit IgG conjugated with Alexa 568 were from Invitrogen. Hoechst dye was purchased from Thermo Fisher Scientific.

### Immunocytochemistry of Cauda Epididymal Sperm

Collected mouse sperm cells were washed two times with PBS and attached on glass coverslips by centrifugation at 700 × *g* for 5 min. Coverslips were fixed with acetone at –20°C for 5 min (DNAH1 and acetylated tubulin), methanol at –20°C for 10 min (γ-tubulin), or 4% paraformaldehyde (PFA) in PBS at room temperature (RT) for 10 min. PFA-fixed coverslips were permeabilized with 0.1% (SEPTIN4, SEPTIN12, and CENTRIN1), 0.2% (DNAH9, and RSPH3), or 1% (DNAH2) Triton X-100 in PBS. Permeabilized coverslips were blocked with 10% normal goat serum in PBS (DNAH1, DNAH2, γ-tubulin, CENTRIN1, SEPTIN4, SEPTIN12, RSPH3, and acetylated tubulin) or 5% normal goat serum with 0.2% Triton X-100 in PBS (DNAH9) at RT for 1 h. Blocked coverslips were incubated with primary antibodies, α-DNAH1 (3 μg/ml), α-DNAH2 (0.5 μg/ml), α-DNAH9 (5 μg/ml), α-RSPH3 (10 μg/ml), α-SEPTIN4 (1 μg/ml), α-SEPTIN12 (1:100), α-γ-tubulin (1:400), α-CENTRIN1 (1:100), or α-acetylated tubulin (1:100) in each blocking buffer at 4°C for overnight. The coverslips incubated with primary antibodies were washed with 0.05% (DNAH9) or 0.1% (DNAH2) Triton X-100 in PBS three times or 0.1% Triton X-100 in PBS one time and PBS two times (acetylated tubulin, γ-tubulin, CENTRIN1, SEPTIN4, SEPTIN12, DNAH1, and RSPH3). The samples were stained with secondary antibodies (1:1,000) in 5% (DNAH9) or 10% (DNAH1, DNAH2, γ-tubulin, CENTRIN1, SEPTIN4, SEPTIN12, RSPH3, and acetylated tubulin) normal goat-serum in PBS for 1 h at RT. Hoechst was used to counterstain sperm nucleus. Immunostained sperm samples were mounted with Vectashield (Vector Laboratories) and imaged with Zeiss LSM710 Elyra P1 using Plan-Apochromat 63X/1.40 oil objective lens.

### Immunocytochemistry of Testicular Germ Cells

Mouse seminiferous tubules were dissociated in ice-cold PBS after removing tunica albuginea. The seminiferous tubules were chopped, vortexed gently to isolate testicular cells followed by filtering in cell strainers with 40 μm pores to collect dissociated testicular germ cells. Collected cells were mixed with PFA in 3% concentration in PBS and attached on glass coverslips coated with poly-D-lysine by centrifuge at 250 × *g* for 5 min. The fixed coverslips were permeablized with 0.1% Triton X-100 in PBS for 10 min, blocked with 10% normal goat serum in PBS for an hour, and incubated with CENTRIN1 (1:100) or SEPTIN4 (1 μg/ml) antibodies in blocking solution at 4°C for overnight. The coverslips were washed with PBS two times and incubated with secondary antibodies and Hoechst in blocking solution for an hour at RT. The immunostained coverslips were mounted and imaged as described in sperm immunocytochemistry.

### RNA Extraction, cDNA Synthesis, and PCR

Total RNA was extracted from WT, *DNAH2*^+/Δ^, and *DNAH2*^Δ/Δ^ testes using RNeasy mini-kit (QIAGEN). One microgram of total RNA was used to synthesize cDNA samples using iScript cDNA Synthesis kit (Bio-Rad). The cDNAs were applied for PCR using OneTaq^®^ 2X Master Mix (NEB) or quantitative PCR using iTaq Universal SYBR Green Supermix (Bio-Rad). Primer pairs used for the RT-PCR were listed in Supplementary Table 2. PCR products were gel-extracted and Sanger sequenced.

### Scanning Electron Microscopy

Sperm cells were attached on the glass coverslips and fixed with 2.5% glutaraldehyde (GA) in 0.1M sodium cacodylate buffer (pH 7.4) for 1 h at 4°C and post fixed in 2% osmium tetroxide in 0.1M cacodylate buffer (pH 7.4). The fixed samples were washed with 0.1M cacodylate buffer for three times and dehydrated through a series of ethanol to 100%. The samples were dried using a 300 critical point dryer with liquid carbon dioxide as transitional fluid. The coverslips with dried samples were glued to aluminum stubs and sputter coated with 5 nm platinum using a Cressington 208HR (Ted Pella) rotary sputter coater. Prepared samples were imaged with Hitachi SU-70 scanning electron microscope (Hitachi High-Technologies).

### Transmission Electron Microscopy

Collected epididymal sperm cells were washed and pelleted by centrifugation and fixed in 2.5% GA and 2% PFA in 0.1M cacodylate buffer pH 7.4 for 1 h at RT. Fixed sperm pellets were rinsed with 0.1M cacodylate buffer and spud down in 2% agar. The chilled blocks were trimmed, rinsed in the 0.1M cacodylate buffer, and replaced with 0.1% tannic acid in the buffer for 1 h. After rinsing in the buffer, the samples were post-fixed in 1% osmium tetroxide and 1.5% potassium ferrocyanide in 0.1M cacodylate buffer for 1 h. The post-fixed samples were rinsed in the cacodylate buffer and distilled water, followed by en bloc staining in 2% aqueous uranyl acetate for 1 h. Prepared samples were rinsed and dehydrated in an ethanol series to 100%. Dehydrated samples were infiltrated with epoxy resin Embed 812 (Electron Microscopy Sciences), placed in silicone molds and baked for 24 h at 60°C. The hardened blocks were sectioned in 60 nm thickness using Leica UltraCut UC7. The sections were collected on grids coated with formvar/carbon and contrast stained using 2% uranyl acetate and lead citrate. The grids were imaged using FEI Tecnai Biotwin Transmission Electron Microscope (FEI, Hillsboro, OR, United States) at 80 kV. Images were taken using MORADA CCD camera and iTEM (Olympus) software.

### Statistical Analysis

Statistical analysis was performed with Student’s *t*-test. Differences were considered significant at ^∗^*p* < 0.05; ^∗∗^*p* < 0.01; ^∗∗∗^*p* < 0.001.

## Results

### Clinical Assessment of the Infertile Males in a Consanguineous Family From Pakistan

A consanguineous family with infertile males was recruited from Pakistan. All the infertile males in this study failed to conceive over 10 years of marriages and unprotected sex (Table 1). No PCD-related symptoms were reported by the infertile males. They exhibited normal heights, weights, and secondary characteristics, were not obese, and were free of tuberculosis as children. They had no anatomic defects, ejaculatory failure, or immunological problems, and experienced no environmental exposure to chemical or radioactive elements. Their karyotypes are normal, and they carry no Y chromosome microdeletions. The follicle-stimulating hormone (FSH), luteinizing hormone (LH), prolactin, and testosterone were within normal range in all affected males.

**TABLE 1:** Clinical diagnosis of the infertile patients.

	Normal range	Units	IV-1	IV-3	V-1
Age of patients			50	43	28
Years of marriages			30	23	10
**Semen Parameters**					
Semen volume	>1.5	ml	3.2	3.7	4
Semen pH	Alkaline		Alkaline	Alkaline	Alkaline
Sperm concentration (10^6^/ml)	>15	ml	10	9	7
Morphologically normal sperm	>4	(%)	20	18	8
Motility (a+b+c)	>40	(%)	14	20	10
Progressive motility (a+b)	>32	(%)	4	7	0
Rapid progressive (WHO a)		(%)	1	3	0
Slow progressive (WHO b)		(%)	3	4	0
Non progressive (WHO c)		(%)	10	13	10
Immotile (WHO d)		(%)	86	80	90
**Hormones**					
FSH	1.7–11.2	mlU/mL	6.5	7.7	5.6
LH	2.1–18.6	mlU/mL	8.9	10.1	7.8
Prolactin	3.6–16.3	ng/ml	9.4	11.3	8.3
Testosterone	62–870	ng/dL	427.5	507.1	618.2

### A New Variant in *DNAH2* Causes Oligoasthenozoospermia

**FIGURE 1:**
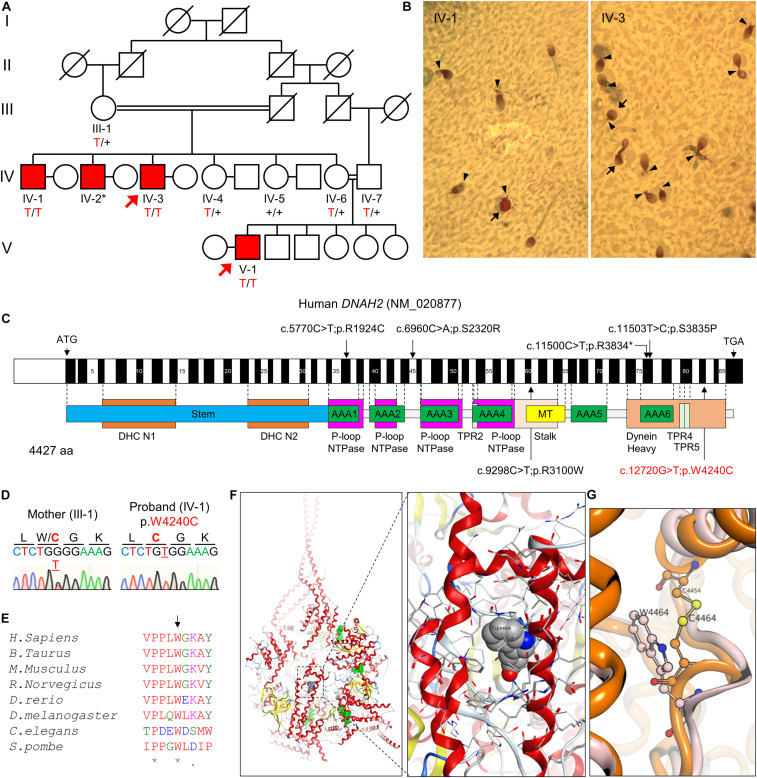
A novel bi-allelic *DNAH2* variant identified from a consanguineous family with oligoasthenozoospermia. **(A)** Pedigree of a consanguineous family with four infertile males (IV-1, IV-2, IV-3, and V-1). WES was performed with IV-3 and V-1 (arrows). Sanger sequencing verified segregation of the variant (red) in the infertile males. A plus (+) indicates a wild-type allele and an asterisk (*) denotes a sample not available for this study. **(B)** PAP-stained semen from patients IV-1 and IV-3. Multiple morphological defects including near absence of a tail or a short tail (arrowheads), and spherical heads (arrows) are prominent from both patients. **(C)** Mapping of the *DNAH2* variant. Mutation of G to T in exon 82 of *DNAH2* cDNA (c.12720G > T; NCBI RefSeq identifier NM_020877) results in a tryptophan-to-cysteine (p.W4240C) substitution in the dynein heavy chain domain. Previously reported pathogenic variants (Li et al., 2019) are also marked (black). **(D)** Chromatograms of the *DNAH2* non-synonymous variant in an infertile sibling (IV-1) and his mother (III-1). The variant is underlined, and the resulting amino acid substitution is marked in red. **(E)** A sequence alignment of the DNAH2 protein across multiple species. The tryptophan at position 4240 (arrow) is conserved in multiple organisms. An asterisk (*) indicates positions fully conserved and a period (.) indicates positions with weakly similar amino acids. **(F,G)** Structural modeling of the DNAH2 mutation from this study. **(F)** Ribbon structure of human DNAH1 (PDB ID: 5NUG), a homolog of human DNAH2. ATP-binding sites are colored in green (*left)*. The enlarged area is the region corresponding to that of W4240 in DNAH2 (*right*). DNAH1 W4464, which is homologous to W4240, is represented in a space-filling model. **(G)** Ribbon diagram shows mutation of tryptophan at position 4464 (W4464, orange) to cysteine (W4464C, pink) is predicted to form disulfide bond with nearby C4454 and distort protein backbone structure.

The pedigree comprises five generations; three males from the fourth generation (IV-1, IV-2, and IV-3) and one male from the fifth generation (V-1) are infertile (Figure 1A). Three affected members (IV-1, IV-3, and V-1) were subjected to clinical analyses and initially diagnosed with oligoasthenozoospermia (OAZ, Table 1). Notably, over 80% of sperm cells from the patients were morphologically abnormal by CASA morphological analysis (IV-1, 80%; IV-3, 82%; V-1, 92%). PAP staining revealed morphological abnormalities prominently in the tail, such as near-absent, short, curved, or coiled forms, which is similar to a representative MMAF phenotype, and mild defects in the head shape (Figure 1B). To elucidate the genetic basis of this phenotype, we performed WES on the proband (IV-3) and on his nephew (V-1). Analyzing WES data estimated inbreeding co-efficient of 10.52% and 19.39% with the longest HBD segments of 33.86 cM and 49.22 cM for the proband and his nephew, respectively. WES analysis identified three rare damaging variants but only homozygous non-synonymous variant (c.12720G > T;p.W4240C) in *DNAH2* co-segregated with the phenotype (Figures 1A,C,D). This variant is predicted as “deleterious” by MetaSVM and absent in all public databases including gnomAD and Browse All Variant Online (Bravo) variant browser^3^ (Table 2). This novel variant was inherited by all affected, a highly unlikely event to occur by chance (odds in favor of linkage 1,000:1, LOD score of 3.0) within the pedigree. CNVs were not detected from XHMM analysis of the WES.

**TABLE 2:** Variant detail in the infertility family.

Gene	*DNAH2*	*FZD3*	*MYDGF*
Genome position (Chr-Position)	17-7735087	8-28384879	19-4670172
Transcript position	NM_020877 c.12720G > T	NM_145866 c.602T > C	NM_019107 c.174+1G > T
Mutation type	Non-synonymous	Non-synonymous	Splice donor
Mutation	p.W4240C	p.F201S	c.174+1G > T
ClinVar Allele ID			
dbSNP			rs745851558
gnomAD MAF			0.0000666
Bravo MAF			0.0000319
MetaSVM	D	D	
SIFT	D	D	
PolyPhen	D	D	
CADD	31	26.2	24.4
GTEx TPM in testis*	8.12	5.64	95.44
GTEx Pext in testis^#^	0.41	1	1
Cosegregation	Yes	No	No

*GTEx TPM: The Genotype-Tissue Expression (GTEx) project V7, Transcripts Per Million. ^#^GTEx Pext: GTEx V7, Proportion Expression across Transcripts.

Human DNAH2 is a paralog of DNAH1 (Kollmar, 2016), the nearest known structure dynein (PDBid:5NUG). The DNAH2 variant in our study encodes a residue residing at the dynein heavy domain at C-terminus, which is highly conserved from human to yeast (Figures 1C,E). Structural modeling based on human DNAH1 demonstrates that the DNAH2 mutant residue is in a buried location within the dynein heavy domain (Figure 1F). Replacing tryptophan with cysteine of DNAH1 at position 4464, which corresponds to the identified DNAH2 mutation in this study, is predicted to have two destabilizing effects. The mutation of the large tryptophan to the small cysteine would generate an energetically destabilizing void space. Alternatively, the cysteine would pair with a nearby cysteine at position 4454, distorting the structure (Figure 1G). Taken together, these data suggest the identified novel *DNAH2* variant is pathogenic and causes male infertility with MMAF-like phenotypes in human, similar to previously reported *DNAH2* variants (Figure 1C; Li et al., 2019).

### Homozygous *Dnah2* Mutant Male Mice Are Infertile

**FIGURE 2:**
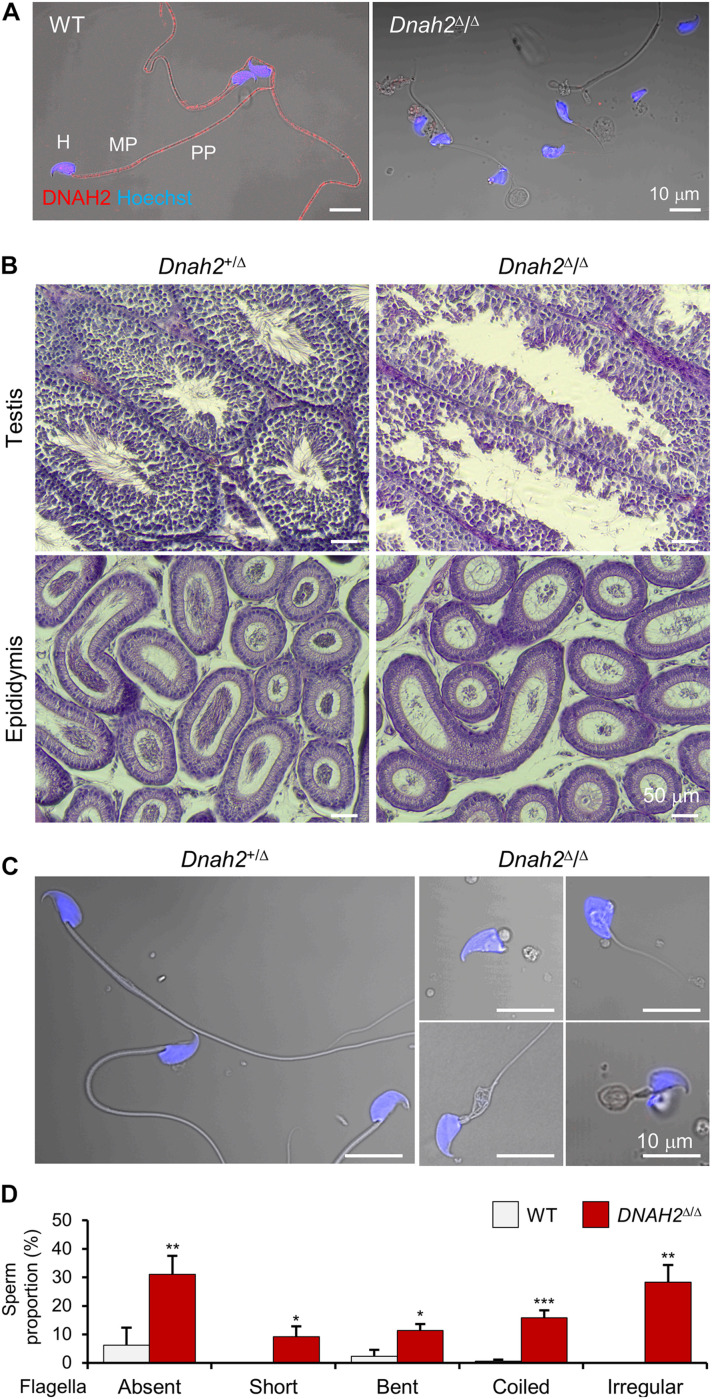
DNAH2 deficiency in mice recapitulates multiple morphological abnormalities of the flagella in humans. **(A)** Confocal images of DNAH2 in WT and *Dnah2*^Δ/Δ^ sperm. DNAH2 were immunodetected from WT sperm but not from *Dnah2*^Δ/Δ^ sperm. Sperm heads were counterstained with Hoechst. Shown images are confocal images merged to the corresponding DIC images. H, head; MP, midpiece; PP, principal piece. **(B)** Testicular and epididymal histology of *Dnah2*^Δ/Δ^ mouse testis and epididymis. H/E stained sections show *Dnah2*^Δ/Δ^ males produce less sperm with elongated tails in the lumen of seminiferous tubule in testis (*top*), resulting in fewer sperm cells in the epididymis (*bottom*, corpus). **(C,D)** Multiple morphological defects in epididymal *Dnah2*^Δ/Δ^ sperm. **(C)** Morphology of *Dnah2*^+/Δ^ (*left*) and *Dnah2*^Δ/Δ^ (*right*) sperm. Sperm from heterozygous males are morphologically normal. *Dnah2*^Δ/Δ^ sperm, however, present MMAF-like phenotypes, such as absent, short, coiled, and irregular-caliber flagella (from *left top*, clockwise direction). Sperm heads were Hoechst-stained (blue). Fluorescence confocal and corresponding DIC images are merged. **(D)** Proportions of sperm with defective flagellar morphologies. Sperm with absent, short, bent, coiled, and irregular-caliber flagella were quantified from WT (gray) and *Dnah2*^Δ/Δ^ (red) male mice (*N* = 3, each). Sperm proportions of each pattern were statistically compared between WT and *Dnah2*^Δ/Δ^ males. Data is represented as mean ± SEM. *p < 0.05; **p < 0.01; ***p < 0.001.

Mutations of genes which encode axonemal IDA, ODA, and RS proteins can cause MMAF (Toure et al., 2020). Infertile males carrying the bi-allelic *DNAH2* variant described in this study also produce sperm with MMAF-like phenotypes (Figure 1B). Currently 39 *DNAH2* SNV are annotated in ClinVar (Supplementary Table 3). Only five *DNAH2* variants, however, were reported to be pathogenic and cause male infertility with MMAF phenotypes in human (Li et al., 2019). To directly demonstrate the genetic causality and elucidate loss of DNAH2 function in general, we characterized *Dnah2* mutant mice. These mice lack the *Dnah2* genomic region spanning exon 25–28, which is predicted to result in a frameshift and early termination of translation (Supplementary Figures 1A–C). RT-PCR result demonstrates that the truncated *Dnah2* mRNAs lacking exon 25–28 are present in testes from homozygous mutant males (Supplementary Figure 1D). Yet the mutant mRNA expression is at about 50% level compared to the total *Dnah2* transcripts in heterozygous mutant males (*Dnah2*^+/Δ^) (Supplementary Figure 1E). These results suggest that *Dnah2*^Δ/Δ^ males cannot generate full-length DNAH2 but potentially a reduced degree of truncated proteins at the C-terminus. Consistently, we validated the absence of the full-length DNAH2 protein in *Dnah2*^Δ/Δ^ sperm by DNHA2 antibody recognizing a C-terminal region located after the deletion (Figure 2A and Supplementary Figure 1F). Both *Dnah2*^+/Δ^ and *Dnah2*^Δ/Δ^ mice showed no gross abnormalities in appearance or survival. *Dnah2^+/Δ^* males and females, and *Dnah2*^Δ/Δ^ females are fertile when mated with WT animals. Homozygous *Dnah2* mutant males, however, are sterile despite their normal sexual behavior. Histological analyses of ciliated epithelia of trachea and oviduct epithelia did not show significant morphological defects in *Dnah2*^Δ/Δ^ mice (Supplementary Figure 2). These results demonstrate that homozygous *Dnah2* mutations cause male-specific infertility in mice.

### *Dnah2*^Δ/Δ^ Males Present MMAF Phenotypes

To elucidate the etiology of infertility of *Dnah2*^Δ/Δ^ males, we examined the histology of *Dnah2*^Δ/Δ^ testis and mutant sperm morphology (Figure 2). Compared to heterozygous mutants, homozygous *Dnah2* mutants had fewer sperm cells in the lumen of seminiferous tubules and epididymis (Figure 2B). Sperm cells collected from the cauda epididymis of *Dnah2*^Δ/Δ^ males showed severe morphological defects to a varying degree (Figures 2C,D). A majority of the *Dnah2*^Δ/Δ^ sperm flagella were absent (31.1 ± 6.5%), short (9.2 ± 3.7%), bent (11.4 ± 2.2%), coiled (15.9 ± 2.5%), or had an irregular-caliber shape (28.4 ± 6.0%), a typical feature of MMAF. A very small number of sperm cells had relatively normal-length, though immotile, tails that only vibrated and twitched (Supplementary Video 1). In addition, *Dnah2*^Δ/Δ^ sperm exhibited mild morphological abnormalities on their head. These sperm characteristics are consistent with the sperm seen in our proband with the identified homozygous *DNAH2* mutation (Figure 1). All these results demonstrate that the homozygous *Dnah2* mutation causes MMAF in mice.

### DNAH2 Is Required for Normal Flagellar Assembly, Organization, and Expression of Axonemal Proteins

**FIGURE 3:**
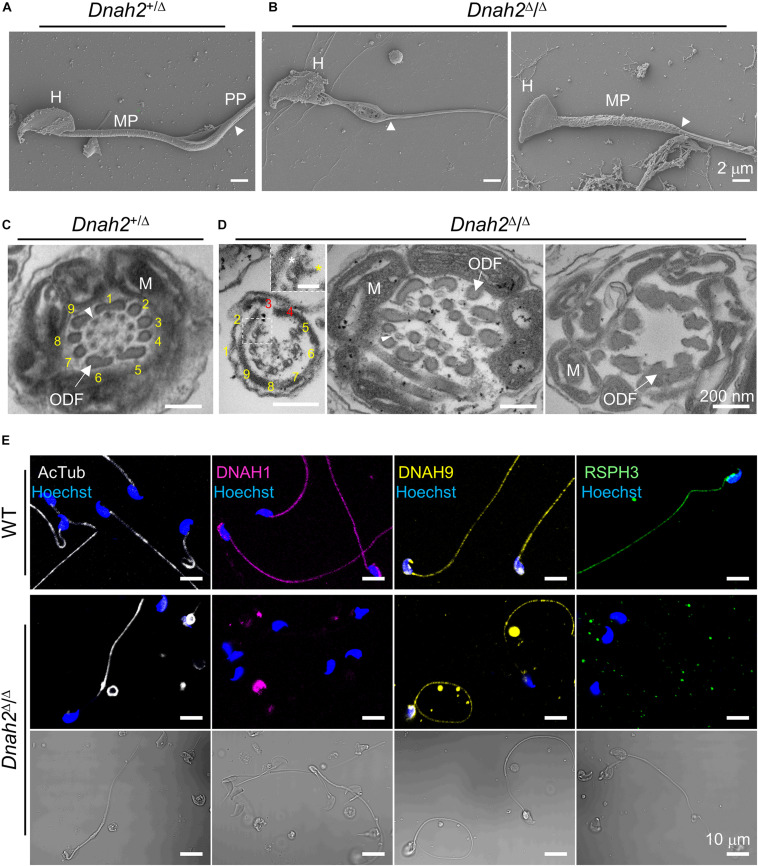
Flagellar ultrastructure is disorganized, and axonemal proteins express abnormally in *Dnah2*-null sperm. **(A,B)** Scanning electron microscopy images of *Dnah2*^+/Δ^ **(A)** and *Dnah2*^Δ/Δ^ **(B)** sperm. *Dnah2*^Δ/Δ^ sperm display absence of (*left*) or an irregular (*right*) mitochondrial helical sheath in the midpiece of the sperm tail, along with abnormal head shapes. Arrowheads indicate annulus. H, head; MP, midpiece; and PP, principal piece. **(C,D)** Transmission electron microscopy images of *Dnah2*^+/Δ^ **(C)** and *Dnah2*^Δ/Δ^ **(D)** sperm. Representative cross-section images of TEM reveal conformational defects in the midpiece of *Dnah2*^Δ/Δ^ sperm. Incomplete 9+2 axonemal structure (*left*), delocalized outer dense fibers (ODF, arrow) and microtubule doublet (*middle*, arrowhead), and lack of the axonemal components (*right*) were observed in the *Dnah2*^Δ/Δ^ sperm. Inner (yellow asterisk) and outer (white asterisk) dynein arm structure were observed from microtubule doublet occasionally (*left*, inset; scale bar = 50 nm). Microtubule doublets are numbered with the absence of the corresponding microtubule doublet in red. M, mitochondria. **(E)** Confocal fluorescence images of axonemal proteins in WT and *Dnah2*^Δ/Δ^ sperm. *Dnah2*^Δ/Δ^ sperm do not express another IDA component (DNAH1, magenta) and a radial spoke protein (RSPH3, green) but an ODA protein (DNAH9, yellow). Flagella microtubules (AcTub, white) and sperm heads (Hoechst, blue) serve as controls. The corresponding DIC images are shown together for *Dnah2*^Δ/Δ^ sperm.

The morphological abnormality led us to examine sperm ultrastructure and molecular organization of flagella (Figure 3 and Supplementary Figure 3). A scanning electron microscopy (SEM) analysis of *Dnah2*^Δ/Δ^ sperm demonstrated morphological defects in the midpiece (Figures 3A,B and Supplementary Figure 3A). Some *Dnah2*^Δ/Δ^ sperm lack mitochondria completely. Others have irregular mitochondrial arrangement around the flagellum and/or mitochondria that wrapped around the head (Supplementary Figure 3A). Transmission electron microscopy (TEM) images revealed diverse ultrastructural defects in the *Dnah2*^Δ/Δ^ flagella (Figures 3C,D and Supplementary Figures 3B–E). Longitudinal section images showed not only abnormally arranged mitochondria, but also misaligned outer dense fiber (ODF) and microtubule doublets in the midpiece region of *Dnah2*^Δ/Δ^ sperm (Supplementary Figures 3B,C). It was frequently observed that the longitudinal columns of the fibrous sheath are not fused to the ODFs at positions 3 and 8 or the whole fibrous sheath is missing in the prospective principal piece (Supplementary Figure 3E). Electron densities corresponding to both IDA and ODA were observed from the microtubule doublets when the 9+2 arrangement is incomplete in the *Dnah2*^Δ/Δ^ axoneme (Figure 3D). However, even mutant sperm with relatively normal morphology cannot beat properly, but only vibrate (Supplementary Video 1), suggesting axonemal dysfunction in IDAs, ODA, and/or RSs. We hypothesized molecular organization of axoneme is disrupted in sperm lacking DNAH2. Another IDA component, DNAH1, is also absent in the flagella of *Dnah2*^Δ/Δ^ sperm (Figure 3E), suggesting that DNAH2 interacts with other IDA components during axonemal assembly. In addition, an RS protein, RSPH3, is also absent in sperm lacking DNAH2, indicating its functional association with RS protein localization. By contrast, *Dnah2*^Δ/Δ^ sperm cells express acetylated tubulin and DNAH9 on their flagella, indicating DNAH2 deficiency might not affect molecular organization of microtubule doublets and ODA. These results demonstrate that the absence of DNAH2 causes abnormal axonemal structure by disrupting molecular organization of not only IDA but also RS and overall organization defects in flagellar development, underlying MMAF-like sperm phenotypes and male infertility.

### DNAH2 Deficiency Dysregulates Developmental Expression and Localization of Basal Body and Annulus Components

**FIGURE 4:**
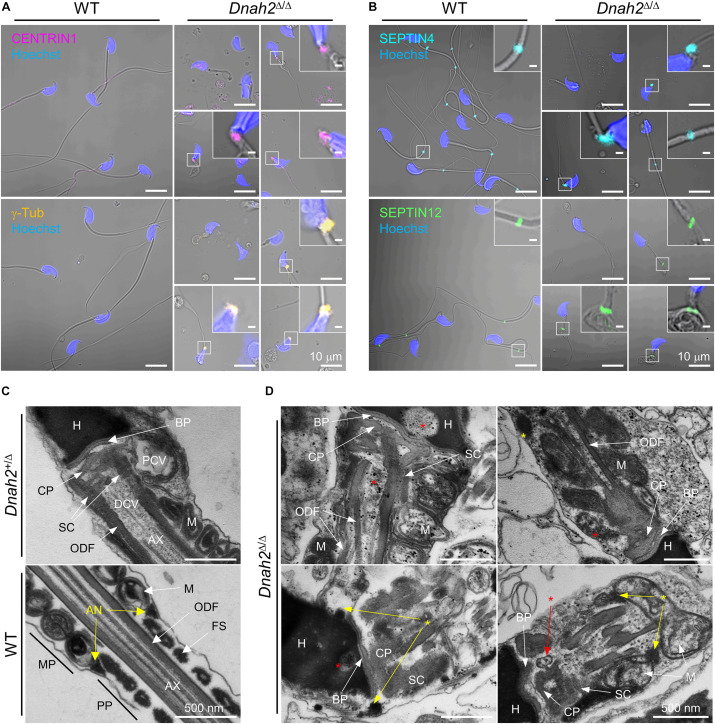
Formation of the basal body and annulus is deregulated in *Dnah2*^Δ/Δ^ epididymal sperm. **(A,B)** Confocal images of immunostained centriolar **(A)** and annulus **(B)** proteins in WT (*left*) and *Dnah2*^Δ/Δ^ (*right*) epididymal sperm. Centriolar proteins, CENTRIN1 (*top*) and γ-Tubulin (*bottom*), lacking in mouse epididymal sperm, are aberrantly detected near the connecting piece of *Dnah2*^Δ/Δ^ sperm **(A)**. Annulus components, SEPTIN4 (*top*) and SEPTIN12 (*bottom*), is localized heterogeneously along the flagella in *Dnah2*^Δ/Δ^ sperm **(B)**. Hoechst was used for counter staining. Merged fluorescence and corresponding DIC images are shown **(A,B)**. Magnified insets are represented (scale bars = 1 μm). **(C,D)** Transmission electron microscopy of normal **(C)** and *Dnah2*^Δ/Δ^ **(D)** epididymal sperm. *Dnah2*^+/Δ^ (*top*) and WT (bottom) sperm were used for controls to show ultrastructure of connecting piece lacking centrioles and annulus, respectively **(C)**. Centriole-like structure or the traces are detected (red asterisks) and annulus-like electron-dense area (yellow asterisks) is localized heterogeneously near the connecting piece in *Dnah2*^Δ/Δ^ sperm **(D)**. Nu, nucleus; BP, basal plate; CP, capitulum; SC, segmented column; PCV, proximal centriolar vault; DCV, distal centriolar vault; AN, annulus; AX, axoneme; M, mitochondria; ODF, outer dense fiber; MP, midpiece; PP, principal piece.

**FIGURE 5:**
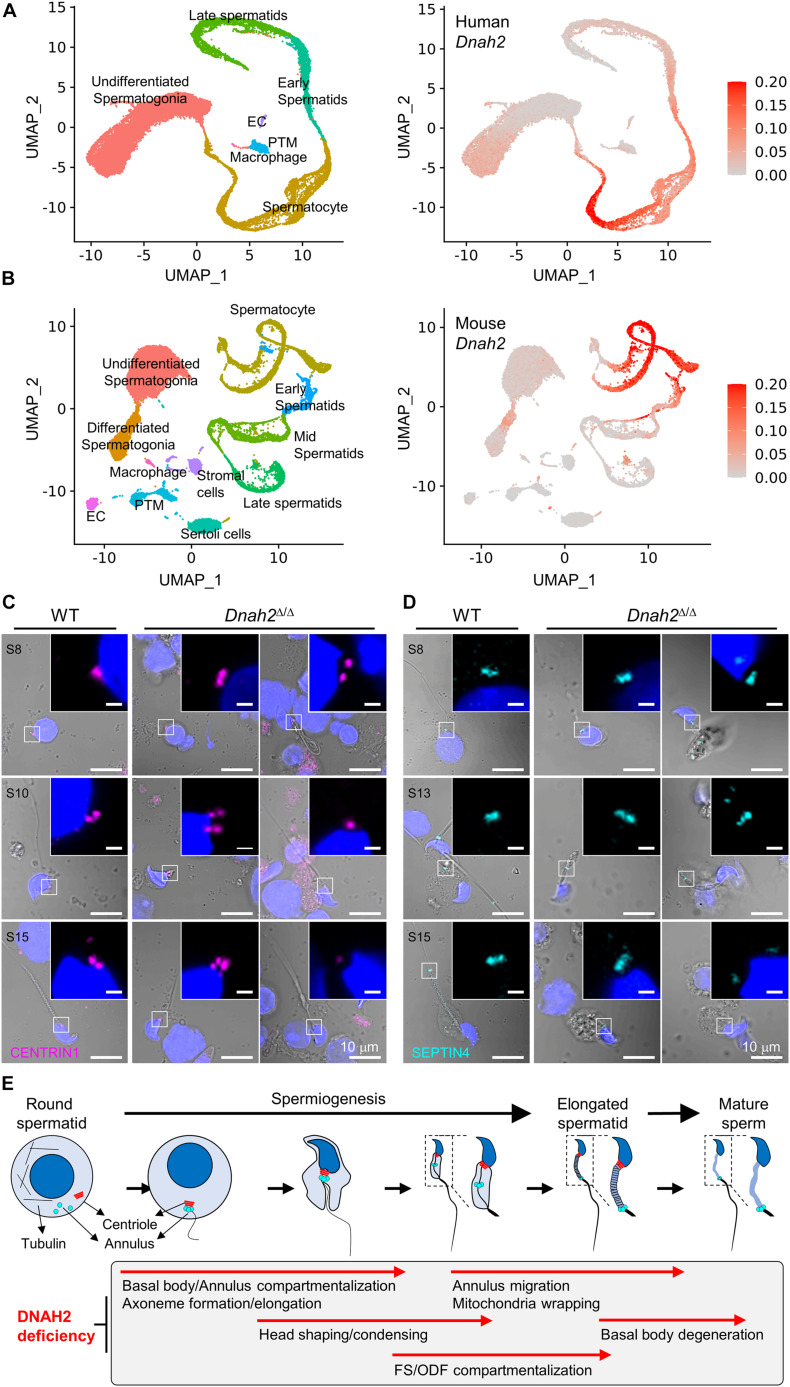
DNAH2 deficiency dysregulates the expression and localization of the basal body and annulus components in developing spermatids. **(A,B)** *DNAH2* mRNA expression in human **(A)** and mouse **(B)** testicular cells. Human (GSE109037) and mouse (GSE109033) testis single-cell RNA (scRNA)-seq datasets were analyzed. UMAP plots represents 7 and 11 clusters of testicular cells in human and mouse (*left*) and *DNAH2* mRNA expression in individual cells (*right*). EC, endothelial cell; PTM, peritubular myoid cell. **(C,D)** Confocal images of centriole (CENTRIN1, **C**) and annulus (SEPTIN4, **D**) proteins in WT and *Dnah2*^Δ/Δ^ spermatids in different developmental step (S). The number of centrioles and localization of the annulus are impaired heterogeneously in *Dnah2*^Δ/Δ^ spermatids. Hoechst was used for counter staining. Merged fluorescence and corresponding DIC images are shown. Magnified insets are shown with fluorescence images (scale bars = 1 mm, **C,D**). **(E)** A schematic cartoon depicting impaired cellular events by DNAH2 deficiency during sperm development. Testicular germ cells from round to elongated spermatids and mature sperm are drawn (*top*) and the corresponding morphological and structural changes are described in a gray box (*bottom*). DNAH2 deficiency compromises flagella elongation and compartmentalization in developing spermatids, which causes in MMAF in mature sperm eventually.

A majority of epididymal sperm from *Dnah2*^Δ/Δ^ males have short flagella (Figure 2), suggesting defects in formation and/or localization of basal body and/or annulus as regulate axoneme elongation and flagellar compartmentalization during spermiogenesis (Avidor-Reiss et al., 2017, 2019). Therefore, we examined expression of centriole and annulus components in epididymal sperm cells (Figures 4A,B). In mice, proximal and distal centrioles to comprise the basal body are degenerated gradually in developing spermatids; in mature epididymal sperm, both proximal and distal centrioles are absent at the connecting piece (Manandhar et al., 2005; Avidor-Reiss et al., 2019). Accordingly, confocal immunostaining revealed that core components of centriole, γ-tubulin and CENTRIN1, are not detected in WT sperm, but remain at connecting piece of *Dnah2*^Δ/Δ^ sperm heterogeneously (Figure 4A). Next, we examined expression of SEPTIN4 and SEPTIN12, components of the annulus, which compartmentalizes sperm flagella (Avidor-Reiss et al., 2017). SEPTIN4 and SEPTIN12 localize at the junction between the midpiece and principal piece in WT sperm, but their expression and localizations are dysregulated heterogeneously in *Dnah2*^Δ/Δ^ sperm like spermatids (Figure 4B); a majority of *Dnah2*^Δ/Δ^ sperm with short tails express SEPTIN4 and SEPTIN12 near the connecting piece. TEM supports the dysregulated expression and localization of the centriole and/or annulus in *Dnah2*^Δ/Δ^ epididymal sperm (Figures 4C,D). Although the basal plate and capitulum are present, overall ultrastructure near the connecting piece is severely disorganized in *Dnah2*^Δ/Δ^ sperm. Notably, centriole-like structures and/or their traces remain in DNAH2-deficient sperm cells in the cavities, which is likely to correspond to the proximal or distal centriole vaults (PCV and DCV, respectively) in *Dnah2*^+/Δ^ sperm (red asterisks in Figure 4D). In addition, *Dnah2*^Δ/Δ^ sperm display annulus-like electron-dense areas much close to the connecting piece which would normally localize at the junction between mitochondria-concentrating midpiece and principal piece in WT sperm (yellow arrows in Figure 4C); sometimes the annulus-like structure is even placed before mitochondria appear (yellow arrows in Figure 4D). All these results demonstrate that DNAH2 deficiency impairs overall spermiogenesis. We utilized the curated public databases to analyze scRNA-seq datasets in human (GSE109037) and mouse (GSE109033) testes and found that *DNAH2* mRNA expresses the highest in spermatocytes and early spermatids among major cell types in both human and mouse testis (Figures 5A,B), supporting the pivotal role of DNAH2 in spermiogenesis.

To better understand DNAH2 function in flagella elongation, especially positioning the basal body and annulus during spermiogenesis, we examined CENTRIN1 and SEPTIN4 in the developing flagella of testicular spermatids from *Dnah2*^Δ/Δ^ males (Figures 5C,D). Just like *Dnah2*^Δ/Δ^ epididymal sperm, after step 8 testicular *Dnah2*^Δ/Δ^ spermatids exhibited obvious flagellar defects in positioning of CENTRIN1 and SEPTIN4. Both WT and *Dnah2*^Δ/Δ^ spermatids express CENTRIN1 at the junction between nucleus and the elongating tail. However, contrary to a set of paired centrioles to form basal body in WT spermatids, the number of CENTRIN1-containing centrioles varies in *Dnah2*^Δ/Δ^ spermatids (Figure 5C). The annulus reaches its final position in the elongating spermatids as depicted by SEPTIN4 localization from the proximal to distal flagellum while advancing developmental stages in WT males (Figure 5D, *left*). In *Dnah2*^Δ/Δ^ spermatids, however, SEPTIN4 expression and localization is variable (Figure 5D, *right*). All of these results strongly suggest that DNAH2 deficiency dysregulates the basal body organization and the annulus migration in developing spermatids (Figure 5E), proving molecular and cellular mechanisms of MMAF.

## Discussion

### Variants in *DNAH2* Underlying Primary Male Infertility in Human

*DNAH2* encodes a 4,427 amino acid long conserved protein which is an IDA component in the axoneme (Chapelin et al., 1997; Viswanadha et al., 2017). DNAH2 is composed of a microtubule binding domain, ATPase domains (AAA domains) (Figure 1). The AAA domains hydrolyze ATP and enable DNAH2 to regulate beating of motile cilia and sperm flagella as a motor protein (Cho and Vale, 2012; Toure et al., 2020). A previous study identified pathogenic DNAH2 mutations in AAA1 (p.R1924C) and AAA6 domains (p.S3835P and p.R3834^∗^) from MMAF patients (Li et al., 2019). DNAH2 protein levels were reduced in sperm from these patients, and the remaining mutant DNAH2 is likely to interfere with normal DNAH2 function, suggesting important roles for AAA domains in DNAH2 function and stability. The novel *DNAH2* variant identified in this study (NM_020877:c.12720G > T;p.W4240C) localizes at the genomic region encoding C-terminal dynein heavy chain. This domain at C-term of DNAH2 contains the AAA6 domain and structurally retains the ATP binding site without a p-loop motif (pfam03028). Our protein modeling predicts that the identified p.W4240C mutation could alter the ATP binding and ATP hydrolysis ability in the mutant DNAH2, likely causing male infertility with MMAF-like phenotype in these Pakistani patients.

### Loss-of-Function Mutations in *DNAH2*, an Evolutionarily Conserved IDA-Encoding Gene, Cause MMAF in Human and Mouse

*DNAH1* and *DNAH2* are paralogs and are evolutionarily conserved across ciliated eukaryotes. They encode IDA heavy chains in motile cilia and sperm flagella. Previous studies demonstrate pathogenic *DNAH1* or *DNAH2* variants cause male infertility and MMAF without obvious PCD symptoms. Two DNAH1 mutations (p.P3909Rfs^∗^33 and p.G3930Afs^∗^120) at the DHC domain at C-terminus were identified from males with MMAF-phenotypes (Ben Khelifa et al., 2014; Wang et al., 2017), similar to DNAH2 mutations at the corresponding domain (Figure 1; Li et al., 2019). Thus, *DNAH1* and *DNAH2* might coordinate flagella development and movement in humans. By contrast, sperm phenotypes by DNAH1 mutations are different from DNAH2 in mice. DNAH1 deficiency in mice did not induce MMAF (Neesen et al., 2001). *Dnah1*-null males are infertile due to severely reduced motility, but the mutant sperm have normal flagellar ultrastructure. This phenotypic difference illuminates that DNAH2 is more than a component of IDA in mice and is involved in overall sperm flagella development compared to the limited function of DNAH1 to IDA. Taken together, *DNAH2* is an MMAF-causing IDA encoding gene conserved in humans and mice.

### Etiology and Developmental Mechanisms of MMAF Elucidated by *Dnah2* Loss-of-Function in Mice

The dynein arm is typically composed of light, intermediate, and heavy chain proteins (Viswanadha et al., 2017). Currently known MMAF-associated genes encode IDA (DNAH1 and DNAH2) and ODA (DNAH8 and DNAH17) heavy chains and their mutations do not seem to cause PCD (Toure et al., 2020). A previous study revealed that DNAH8 and DNAH17 express specifically in sperm flagella, but the other ODA heavy chains, DNAH5, DNAH9, and DNAH11, express in airway cilia (Whitfield et al., 2019). The protein expression patterns indicate that sperm ODA are composed of specific ODA proteins. MMAF patients with mutations at *DNAH8* and *DNAH17* do not express DNAH17 and DNAH8, respectively. Yet, the ODA ultrastructure observed through TEM in these patients was relatively normal in their sperm (Whitfield et al., 2019; Liu et al., 2020). Similarly, we found that *Dnah2*-null sperm lack both DNAH1 and DNAH2 proteins but retain electron densities corresponding IDA structure (Figure 3 and Supplementary Figure 3). We did not observe a difference in gross morphology of cilia in tracheal or oviductal epithelia from *Dnah2* knockout mice (Supplementary Figure 2). Therefore, it is plausible that DNAH2 functions more specifically in flagella, while DNAH2 contributes to ciliary function awaits further studies.

We found DNAH2 deficiency in mice causes MMAF phenotypes and mildly abnormal heads (Figures 2–4 and Supplementary Figure 3), indicating defects in flagella elongation and head shaping during spermiogenesis. The basal body, originating from mother and daughter centrioles, is a platform for microtubule elongation to form the axoneme in ciliates (Werner et al., 2017). Recent studies identify that mutations of centriolar proteins, DZIP1 and CEP135, from MMAF patients (Sha Y. W. et al., 2017; Lv et al., 2020), highlighting that defective basal body compromises flagellar development. Unexpectedly, we found some *Dnah2*^Δ/Δ^ spermatids have more than two centrioles. Considering that *DNAH2* expresses predominantly in spermatocytes (Figure 5) and that centrosome duplication occurs in spermatocytes (Alfaro et al., 2021), DNAH2 might be involved in basal body formation in earlier steps in male germ cell development. The proximal region of cilium, called the transition zone, is a specific molecular diffusion barrier to restrict protein entry into cilium. The annulus, SEPTIN-complexed flagellar ring structure, functions as a barrier just like the transition zone in cilium (Avidor-Reiss et al., 2017). Notably, the annulus is initially localized close to the proximal region of the elongating flagella and gradually migrates to the distal part. Immunostaining and TEM images reveal that DNAH2 deficiency impairs the annulus migration in spermatids, resulting in heterogeneous localization in the epididymal sperm. Although how the annulus migrates along the flagella is not well characterized, the defective annulus localization suggests the DNAH2 could be a motor protein to play a role in annulus migration in developing spermatids.

Intraflagellar transporters (IFTs) are essential for proper sperm tail assembly as well as head formation (Lehti and Sironen, 2016). The manchette, sheaths of microtubules extending tailward from the nucleus, is involved in the trafficking of Golgi-derived cargos to the flagellum (Kierszenbaum et al., 2011). DNAH2 might associate with the IFTs and the manchette in developing spermatids. Consistent with this idea, recent human genetic and mouse knockout studies have demonstrated that mutations in IFT components (Liu et al., 2017; Zhang S. et al., 2020) and manchette-associated molecules (O’Donnell et al., 2012; Lehti et al., 2013) result in morphological defects in sperm flagella and heads similar to those seen in *Dnah2*-null sperm (Figure 3 and Supplementary Figure 3). In *Dnah2*-null spermatids, IFT and manchette-mediated transport of axonemal and peri-axonemal components to flagella might have been dysregulated, supported by the loss of DNAH1 and RSPH3. This dysregulation would result in cargo accumulation in the cytoplasm of spermatids, resulting in abnormal head morphology, disorganized 9+2 axoneme, and delocalized peri-axonemal components.

In summary, we identified a novel *DNAH2* variant that segregated with male infertility in a consanguineous family of Pakistani origin. Our genetic, clinical and *in silico* analyses in human subjects and loss-of-function studies in a mouse model elucidate the general pathogenic mechanisms of DNAH2 mutations in the flagellar assembly and beating. DNAH2 deficiency in mice causes male-specific infertility resembling MMAF sperm phenotypes, thus directly establishing DNAH2 as a causative gene to MMAF. Moreover, DNAH2 deficiency not only impairs molecular organization of the axoneme but also other sperm flagellar structures, suggesting the involvement of the dynein complex in sperm flagellar assembly. Our study provides new knowledge to clinicians and genetic counselors for understanding the genetic etiology of MMAF and better planning of assisted reproductive technology for male patients experiencing MMAF-related infertility.

## Data Availability Statement

The datasets presented in this study can be found in online repositories. The names of the repository/repositories and accession number(s) can be found below: https://www.ncbi.nlm.nih.gov/gap/?term=phs000744.

## Ethics Statement

The studies involving human participants were reviewed and approved by the Institutional Review Board of Quaid-i-Azam University, Islamabad, Pakistan. The patients/participants provided their written informed consent to participate in this study. The animal study was reviewed and approved by Yale Animal Care and Use.

## Author Contributions

J-JC and WA conceived and supervised the project. JH and HW performed mouse characterization experiments. SN recruited patients and analyzed patient samples with help from SH and MN. SN and J-NO prepared the DNA samples for WES. JC, FL-G, KB, and SM generated WES data. SN, JC, FL-G, and WD analyzed WES data. JC and KJ analyzed scRNA-seq data. CB modeled protein structures. J-JC, WA, C-KL, and RL provided resources. J-JC administrated entire study. JH, SN, JC, and J-JC wrote the manuscript with the inputs from all other authors. All authors contributed to the article and approved the submitted version.

## Conflict of Interest

The authors declare that the research was conducted in the absence of any commercial or financial relationships that could be construed as a potential conflict of interest.

## Footnotes

1 https://gnomad.broadinstitute.org/

2 https://www.ncbi.nlm.nih.gov/geo/

3 https://bravo.sph.umich.edu/freeze8/hg38/about
