# Supplementary material for: Genetic Defects in DNAH2 Underlie Male Infertility With Multiple Morphological Abnormalities of the Sperm Flagella in Humans and Mice
Source: Front Cell Dev Biol. 2021 Apr 23;9:662903. doi: 10.3389/fcell.2021.662903 (PMC8103034; doi:10.3389/fcell.2021.662903)
Supplement: Supplementary file 5 [file Image_1.pdf]

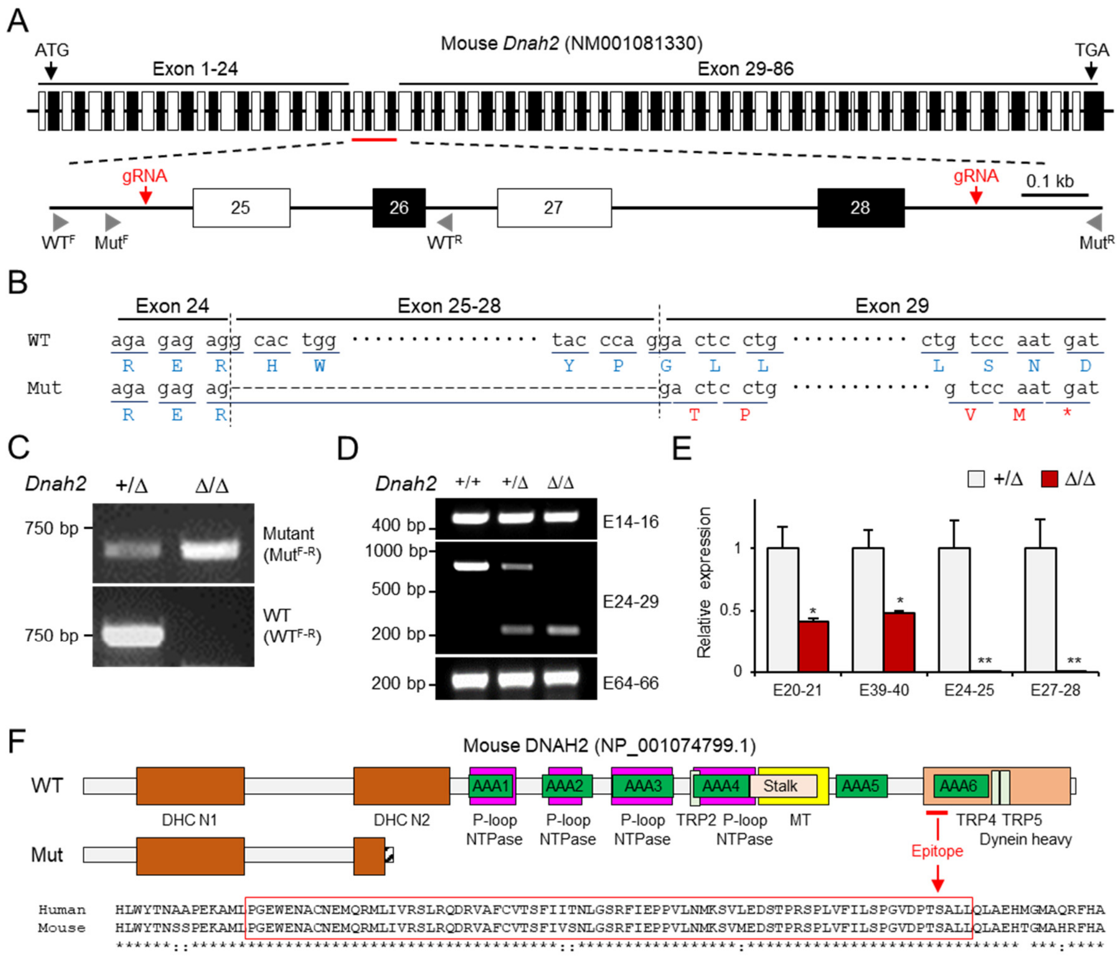

Supplementary Figure 1. *Dnah2*-null mice were generated by CRISPR/Cas9 genome editing. **(A-B)** Genomic structure of mouse *Dnah2* and the CRISPR/Cas9-created mutant allele lacking a region spanning exon 25-28. **(A)** Two guide RNAs targeting introns 24 and 28 were used to generate the mice. Gray arrowheads show the locations of the genotyping primers. **(B)** This mutation is predicted to induce frameshift and early termination of translation. Codons are underlined with the corresponding amino acids. **(C)** Genotyping of *Dnah2*-mutant mice. WT and mutant alleles are detected by genomic DNA PCR with primer pairs marked in panel A. (**D-E**) *Dnah2* mRNA expression in testes from *Dnah2* mutant males. (**D**) Expression of mutant *Dnah2* transcripts with deletion was confirmed by RT-PCR using primers to target the region spanning from exon 24 to 29 (E24-29). The PCR products were Sanger sequenced. (**E**) *Dnah2* expression levels in testis from *Dnah2*$^{+/\Delta}$ (gray) were compared quantitatively to that of *Dnah2*$^{\Delta/\Delta}$ (red) testis. Primer pairs to detect deleted (exon 24 and 25, E24-25; exon 27 and 28; E27-28) or non-deleted (exon 20 and 21, E20-21; exon 39 and 40, E39-40) *Dnah2* mRNA were used. Data is represented

by mean ± SEM. *p<0.05; **p<0.01. **(F)** Domain organizations of mouse WT (top) and mutant DNAH2 (*bottom*). Out-of-frame region in the truncated DNAH2 from mutated allele (Mut) is slashed. The human DNAH2 antibody used in this study recognizes the underlined epitope; the amino acid sequences of the human epitope is aligned to the homologous sequence of mouse DNAH2 (red box).

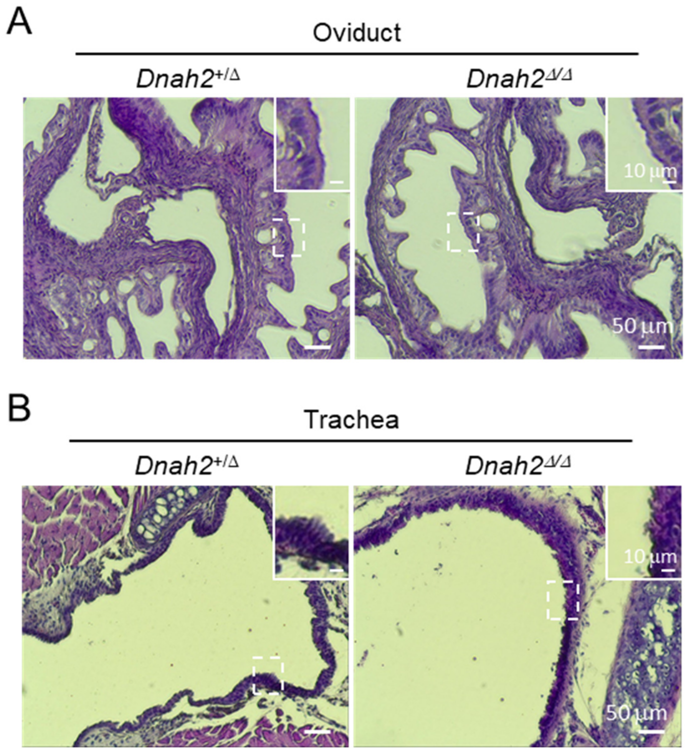

Supplementary Figure 2. Oviductal (**A**) and tracheal histology of (**B**) *Dnah2*$^{+/\Delta}$ (*left*) and *Dnah2*$^{\Delta/\Delta}$ mice (*right*). H/E stained sections are shown. Luminal epithelia in oviduct and trachea are ciliated in both *Dnah2*$^{+/\Delta}$ and *Dnah2*$^{\Delta/\Delta}$ mice. Insets represent magnified view of the ciliated epithelia (*right top*).

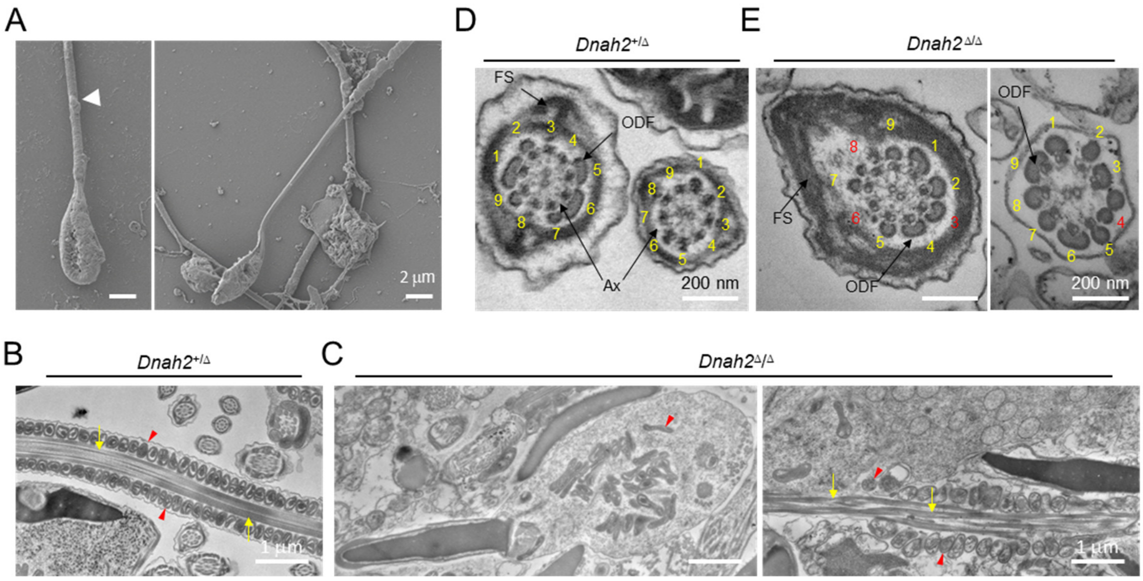

Supplementary Figure 3. Heterogeneous ultrastructure of *Dnah2*$^{Δ/Δ}$ sperm. (**A**) SEM images show the presence (*left*) or absence (*right*) of a mitochondrial sheath in *Dnah2*$^{Δ/Δ}$ sperm. An arrow indicates annulus. (**B-E**) Longitudinal (**B-C**) and transverse (**D-E**) sections of TEM images show morphological defects in *Dnah2*$^{Δ/Δ}$ sperm tails. (**B-C**) *Dnah2*$^{Δ/Δ}$ sperm show delocalized mitochondria (**C**, *left,* arrowheads) and irregular arrangement of outer dense fibers (**C**, *right,* arrow). (**D-E**) The principal piece and endpiece of *Dnah2*$^{Δ/Δ}$ sperm show disarrangement of outer dense fibers (ODFs) and microtubule doublets. Number 6 ODF is missing and number 3 and 8 ODFs are not incorporated into the fibrous sheath of the principal piece (**E**, *left*). Mitochondrial sheath and number 4 microtubule doublet are missing in the flagella of *Dnah2*$^{Δ/Δ}$ sperm (**E**, *right*).

Supplementary Table 1. Sanger sequencing primer pairs.

Supplementary Table 2. Primer pairs for mouse *Dnah2* RT-PCR.

Supplementary Table 3. Reported DNAH2 variants and their pathogenicity in ClinVar database.

Supplementary Video 1. Morphology and motility of *Dnah2*$^{\Delta/\Delta}$ spermatozoa. Sperm released from cauda epididymis was recorded in HS medium at 37 °C; Video rate 100 fps, 4 s movie.
